# Supplementary figures and images for: Transplantation of exogenous mitochondria mitigates myocardial dysfunction after cardiac arrest
Source: eLife. 2025 Apr 10;13:RP98554. doi: 10.7554/eLife.98554 (PMC11984951; doi:10.7554/eLife.98554)

Figure 3—source data 1. PDF file containing uncropped western blots with labeling for panel B.

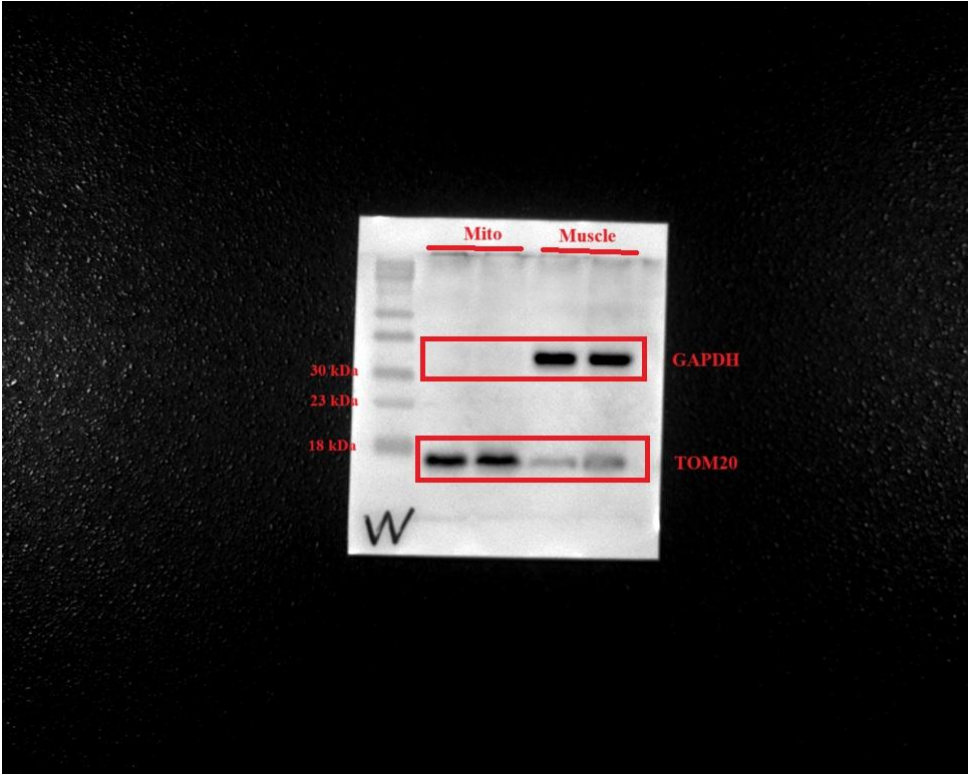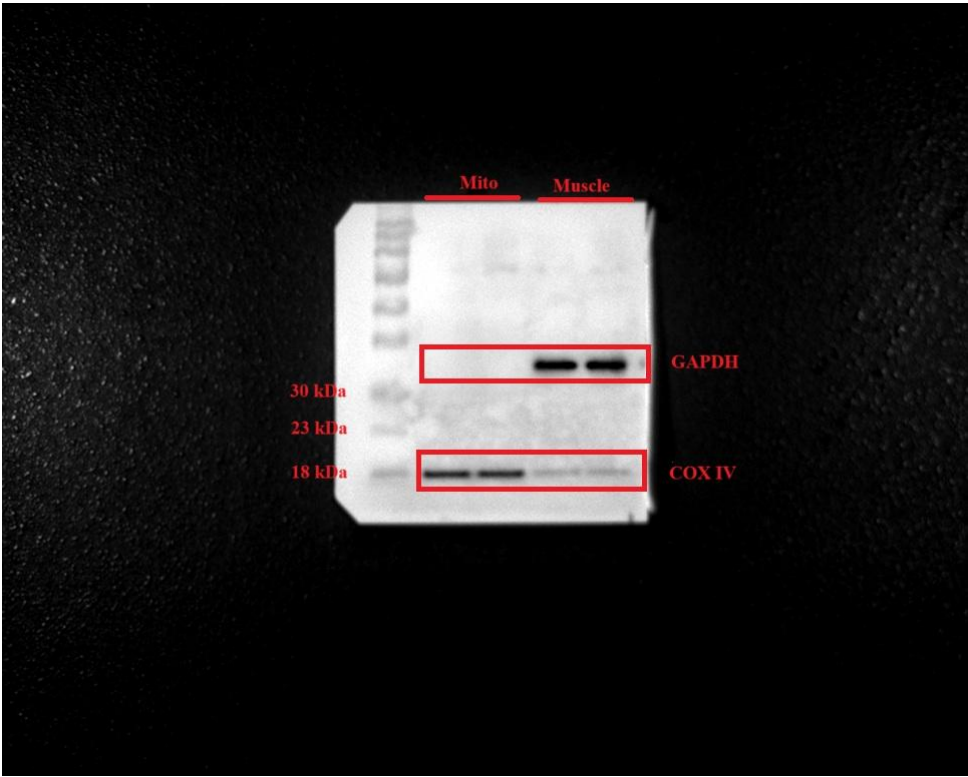

Supplement: Figure 3—source data 1. [file elife-98554-fig3-data1.zip › Figure 3-source data 1/Figure 3—source data 1.pdf]

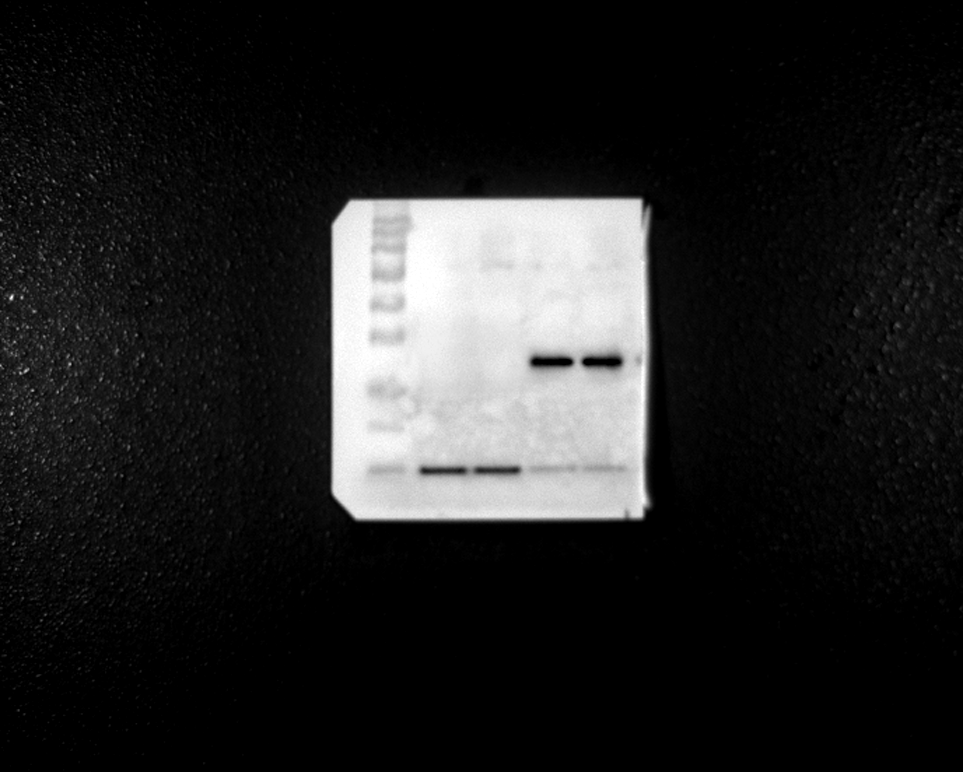

Supplement: Figure 3—source data 2. [file elife-98554-fig3-data2.zip › Fifure3-source data 2/COX IV.tif]

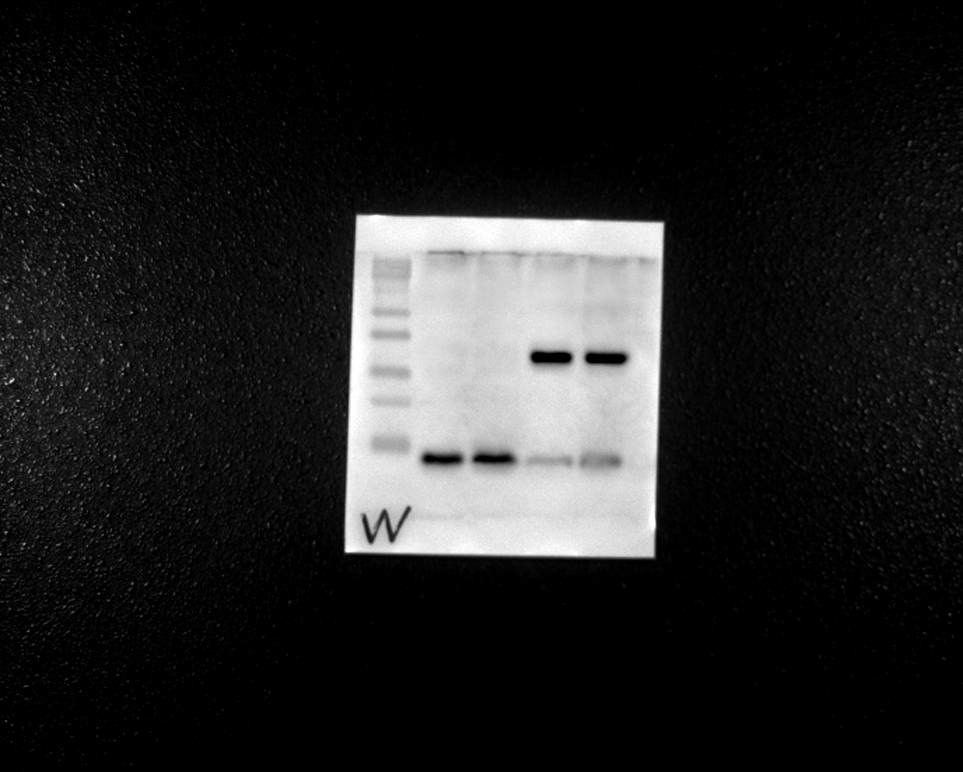

Supplement: Figure 3—source data 2. [file elife-98554-fig3-data2.zip › Fifure3-source data 2/TOM20.tif]

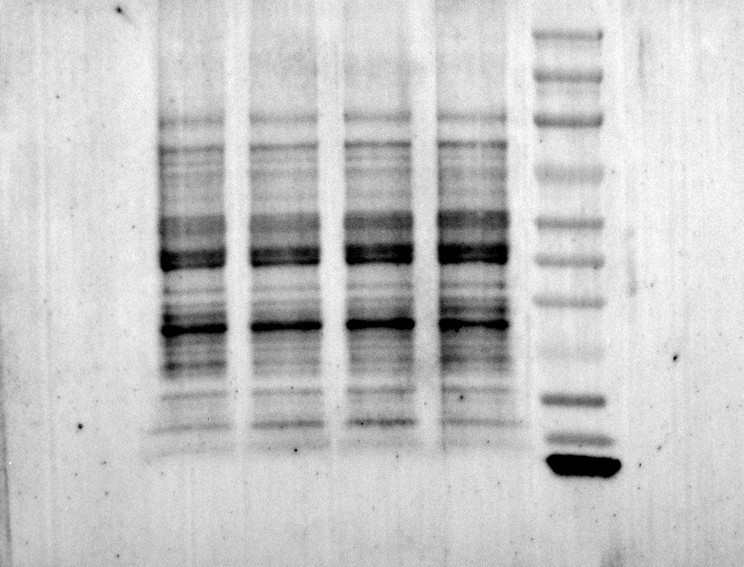

Supplement: Figure 6—source data 2. [file elife-98554-fig6-data2.zip › Figure 6-source data 2/Cleaved-caspase3-1.tif]

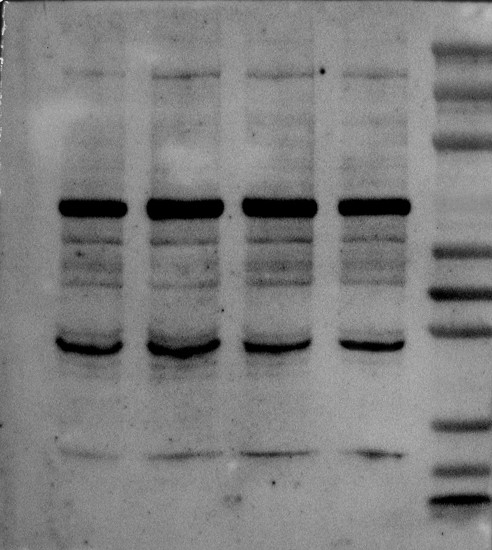

Supplement: Figure 6—source data 2. [file elife-98554-fig6-data2.zip › Figure 6-source data 2/Cleaved-caspase3-2.tif]

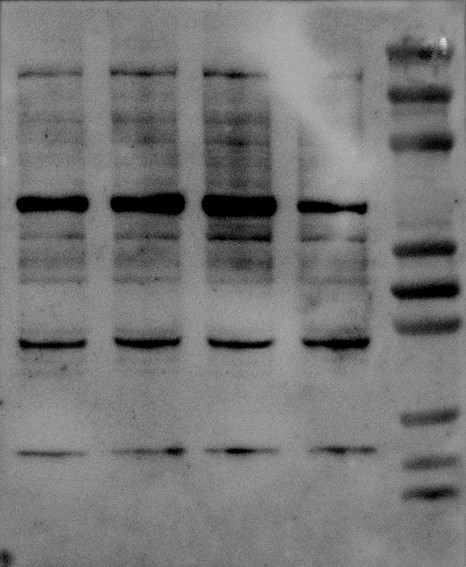

Supplement: Figure 6—source data 2. [file elife-98554-fig6-data2.zip › Figure 6-source data 2/Cleaved-caspase3-3.tif]

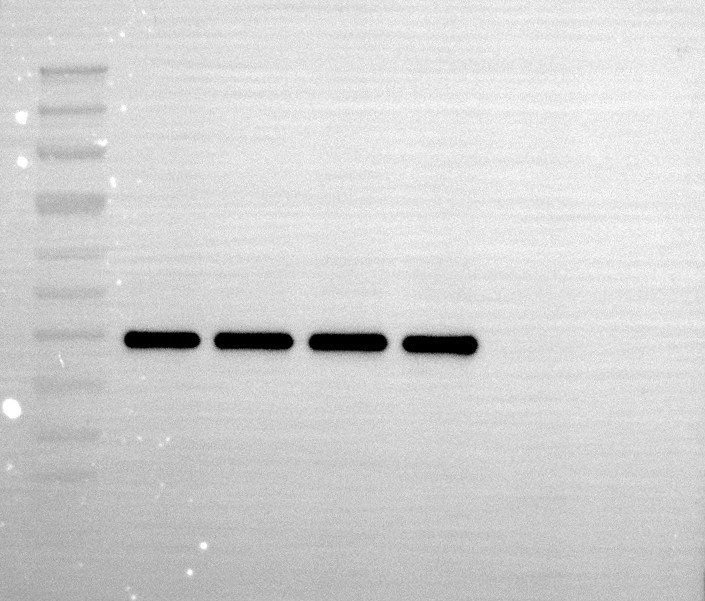

Supplement: Figure 6—source data 2. [file elife-98554-fig6-data2.zip › Figure 6-source data 2/GAPDH-1.tif]

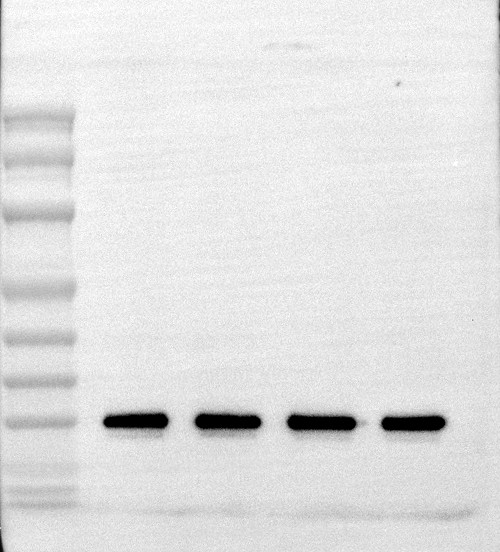

Supplement: Figure 6—source data 2. [file elife-98554-fig6-data2.zip › Figure 6-source data 2/GAPDH-2.tif]

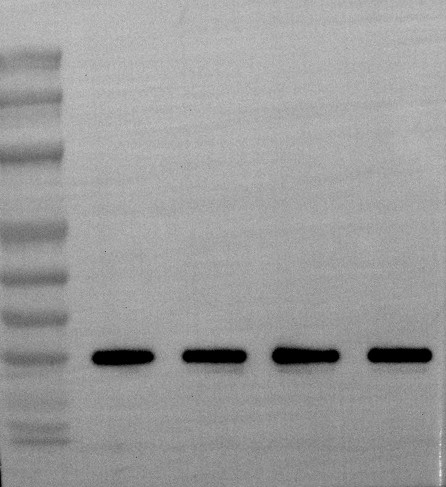

Supplement: Figure 6—source data 2. [file elife-98554-fig6-data2.zip › Figure 6-source data 2/GAPDH-3.tif]
