# Supplementary material for: Transplantation of exogenous mitochondria mitigates myocardial dysfunction after cardiac arrest
Source: eLife. 2025 Apr 10;13:RP98554. doi: 10.7554/eLife.98554 (PMC11984951; doi:10.7554/eLife.98554)
Supplement: Figure 6—source data 1. [file elife-98554-fig6-data1.zip › Figure 6-source data1/Figure 6-source data1.pdf]

Figure 6—source data 1. PDF file containing uncropped western blots with labeling for panel G.

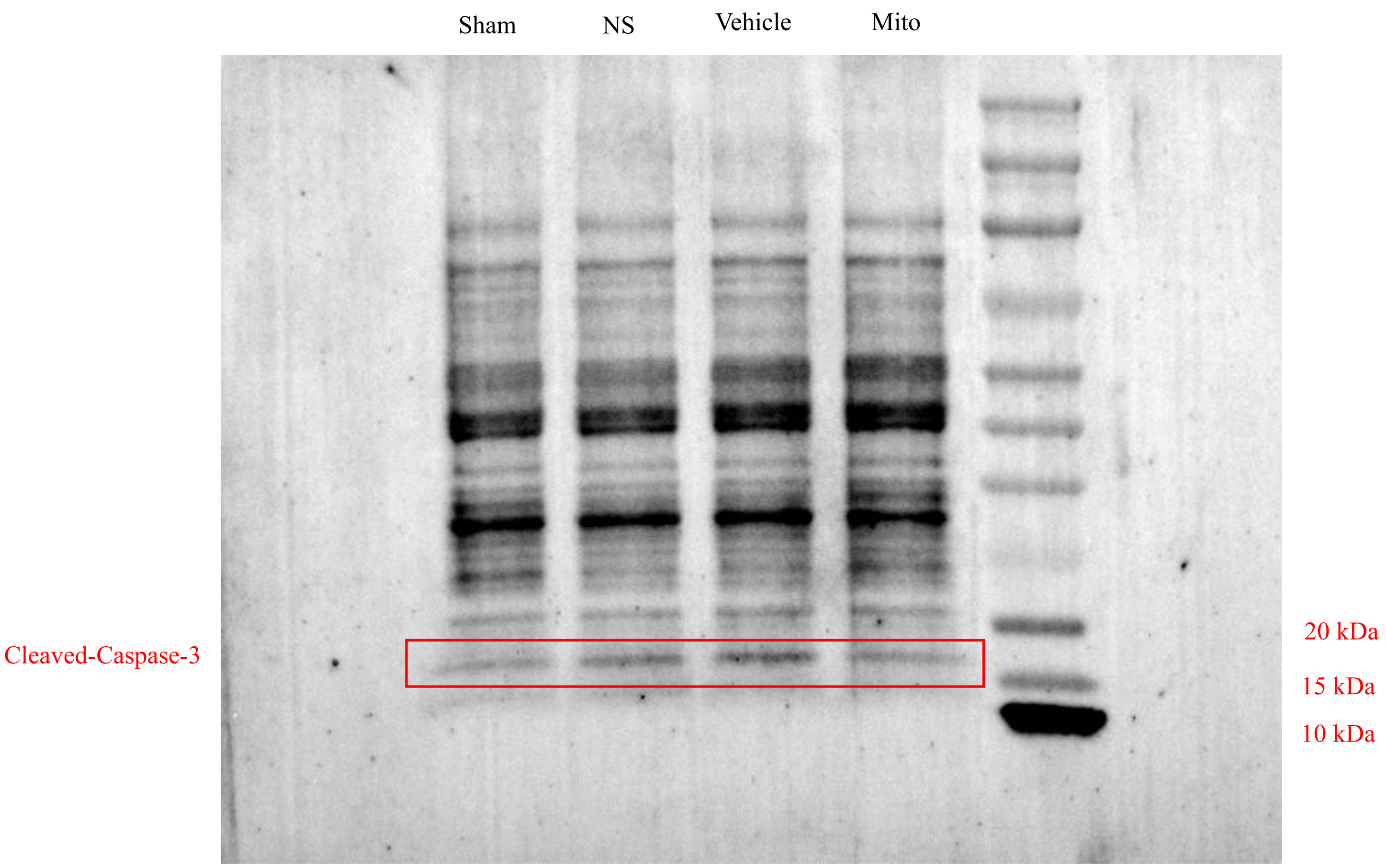

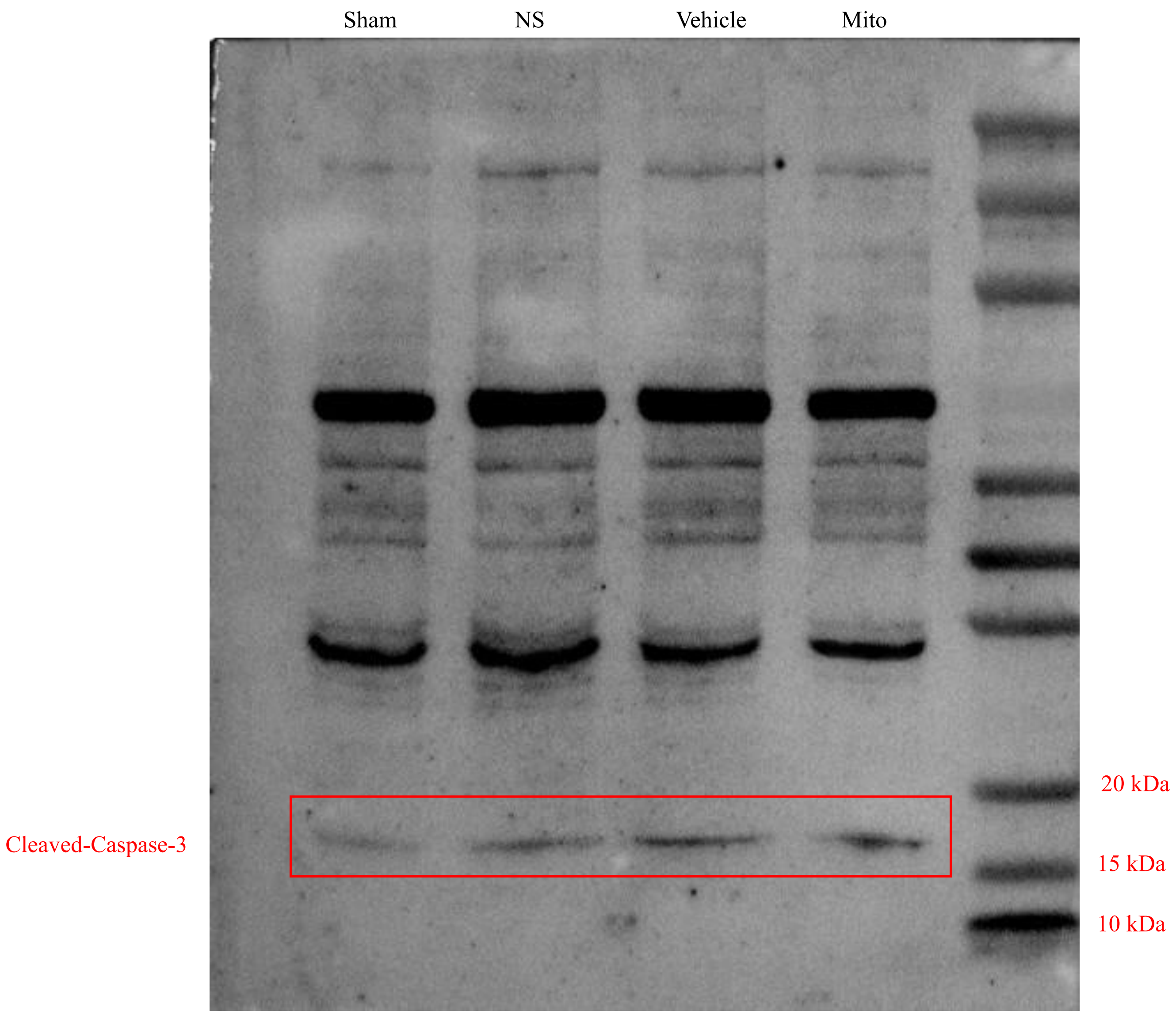
Sham
NS
Vehicle
Mito
Cleaved-Caspase-3
20 kDa
15 kDa
10 kDa

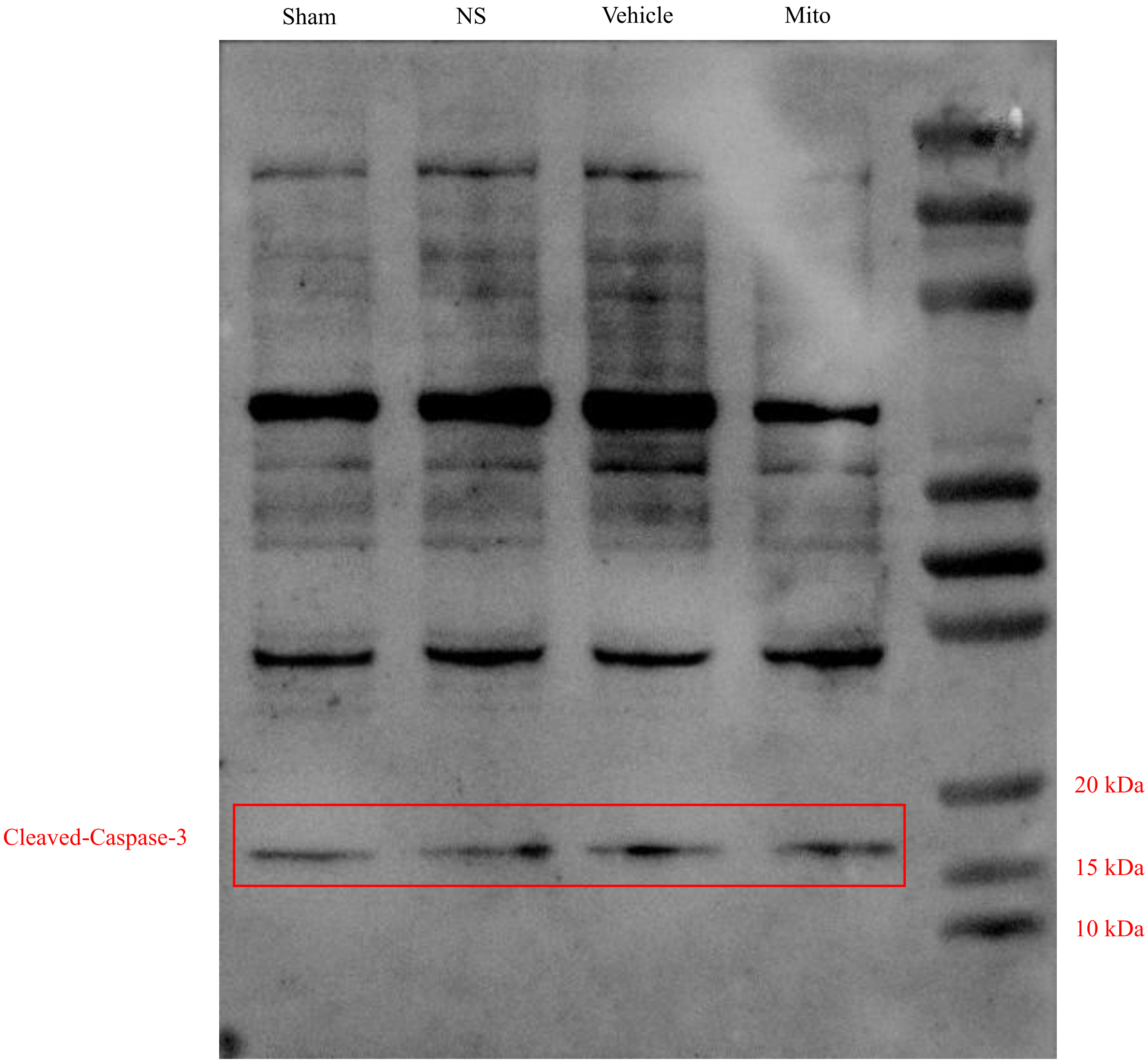
Sham
NS
Vehicle
Mito
Cleaved-Caspase-3
20 kDa
15 kDa
10 kDa

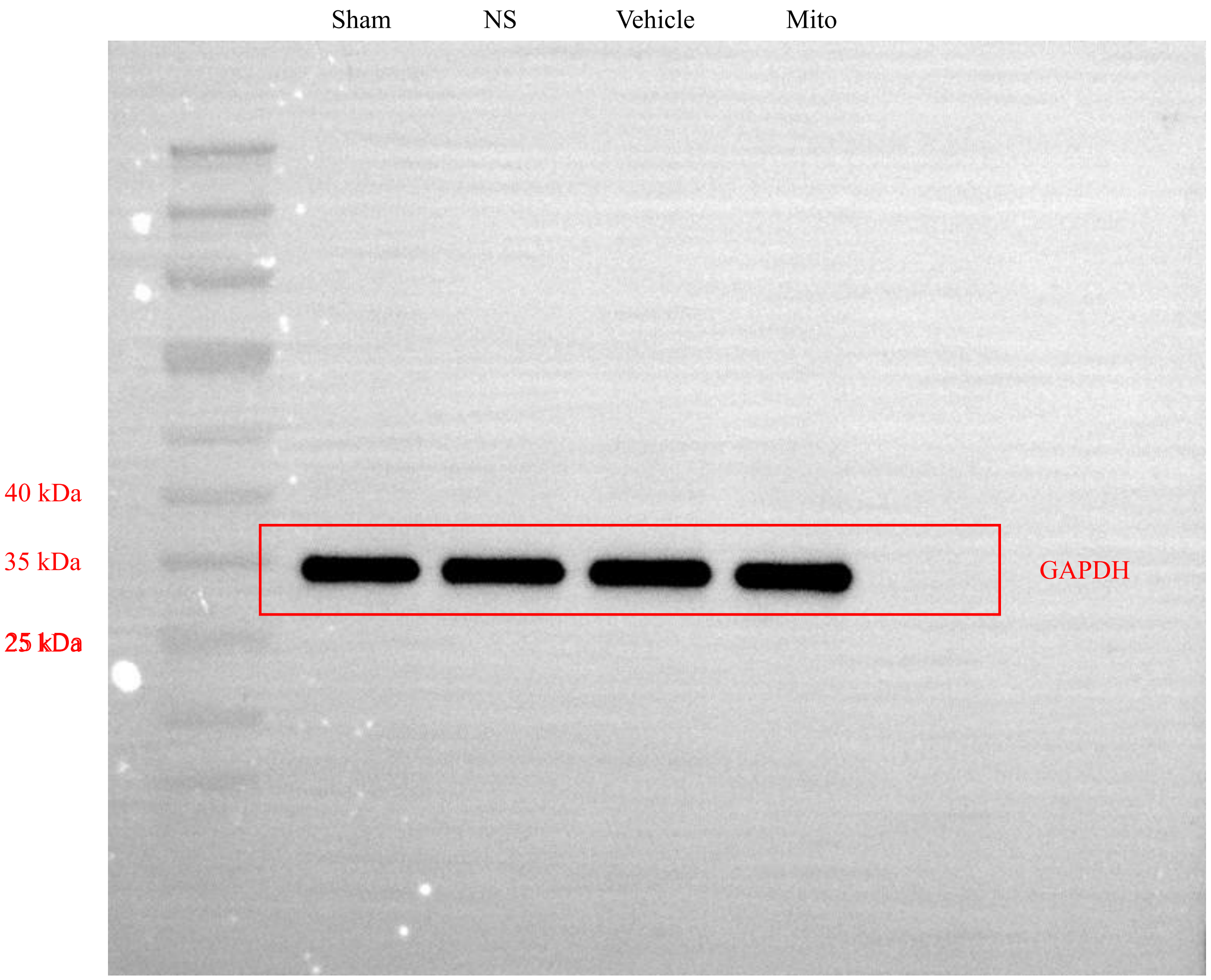
Sham
NS
Vehicle
Mito
40 kDa
35 kDa
25 kDa
GAPDH

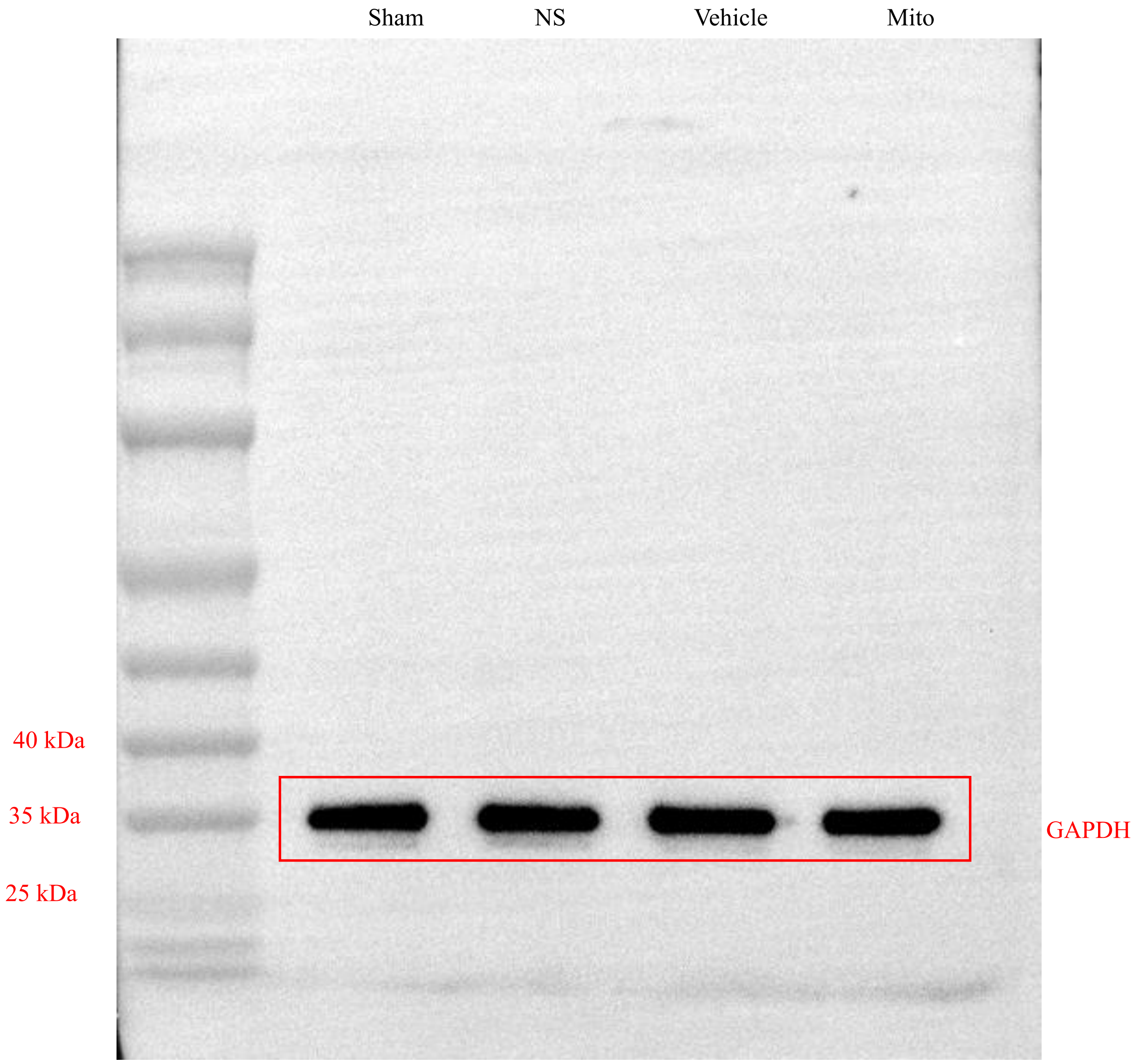
Sham
NS
Vehicle
Mito
40 kDa
35 kDa
25 kDa
GAPDH

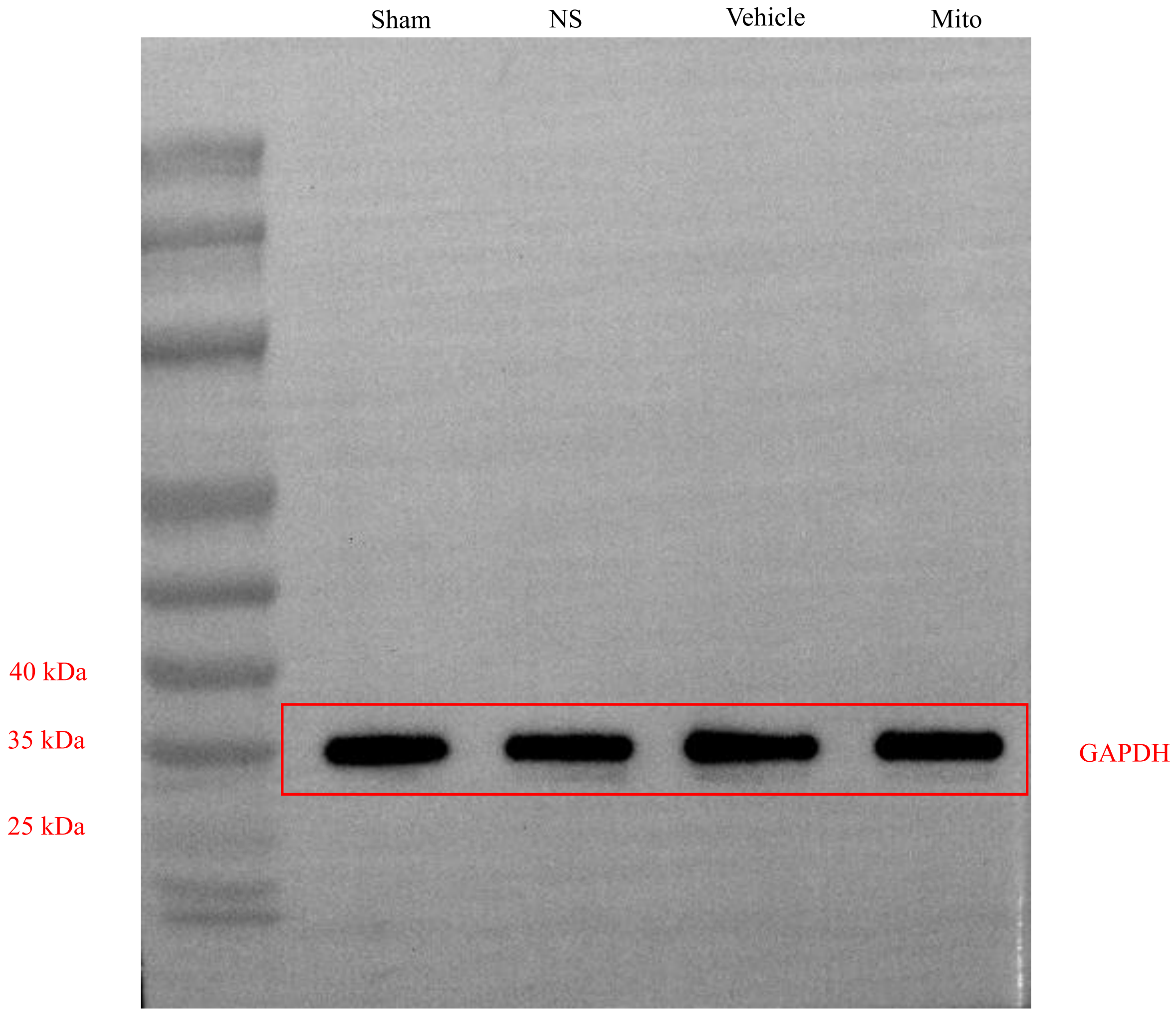
Sham
NS
Vehicle
Mito
40 kDa
35 kDa
25 kDa
GAPDH
