# Supplementary material for: Transplantation of exogenous mitochondria mitigates myocardial dysfunction after cardiac arrest
Source: eLife. 2025 Apr 10;13:RP98554. doi: 10.7554/eLife.98554 (PMC11984951; doi:10.7554/eLife.98554)
Supplement: Supplementary file 2. [file elife-98554-supp2.docx]

**Supplementary File 2** **Scoring Standard of Myocardial Pathological Injury**

| **Grade** | **Scoring Standard** | **Score** |
| --- | --- | --- |
| **0** | The staining of myocardial fiber was homogeneous, cross striation was distinct, myocardial interstitium has no sign of inflammatory cellular invasion, no sign of hemorrhagic necrosis; | 0 |
| **Ⅰ** | Partial myocardial fiber presents wave shape, myoplasm distribution was inhomogeneous, myocardial interstitium has no sign of hemorrhage; | 2 |
| **Ⅱ** | Partial myocardial fiber breakage is noted, with focal hemorrhage, myocardial interstitium has inflammatory cellular hemorrhage; | 4 |
| **Ⅲ** | Partial myocardial has focal necrosis, myocardial interstitium has hemorrhage and inflammatory cellular invasion; | 6 |
| **Ⅳ** | A majority of myocardium has focal necrosis, myocardial interstitium has hemorrhage and inflammatory cellular invasion; | 8 |
| **Ⅴ** | A majority of myocardium has focal necrosis, myocardial interstitium has diffusive hemorrhage and inflammatory cellular invasion. | 10 |
